# Chalcone Derivatives as Potential Inhibitors of P-Glycoprotein and NorA: An *In Silico* and *In Vitro* Study

**DOI:** 10.1155/2022/9982453

**Published:** 2022-03-26

**Authors:** Minh-Tri Le, Dieu-Thuong Thi Trinh, Trieu-Du Ngo, Viet-Khoa Tran-Nguyen, Dac-Nhan Nguyen, Tung Hoang, Hoang-Minh Nguyen, Tran-Giang-Son Do, Tan Thanh Mai, Thanh-Dao Tran, Khac-Minh Thai

**Affiliations:** ^1^Department of Medicinal Chemistry, Faculty of Pharmacy, University of Medicine and Pharmacy at Ho Chi Minh City, 41 Dinh Tien Hoang, Dist 1, Ho Chi Minh City 700000, Vietnam; ^2^School of Medicine, Vietnam National University Ho Chi Minh City, Linh Trung Ward, Thu Duc District, Ho Chi Minh City 700000, Vietnam; ^3^Faculty of Traditional Medicine, University of Medicine and Pharmacy, Ho Chi Minh City 700000, Vietnam; ^4^University Medical Center Ho Chi Minh City, University of Medicine and Pharmacy, Ho Chi Minh City 700000, Vietnam

## Abstract

The human P-glycoprotein (P-gp) and the NorA transporter are the major culprits of multidrug resistance observed in various bacterial strains and cancer cell lines, by extruding drug molecules out of the targeted cells, leading to treatment failures in clinical settings. Inhibiting the activity of these efflux pumps has been a well-known strategy of drug design studies in this regard. In this manuscript, our earlier published machine learning models and homology structures of P-gp and NorA were utilized to screen a chemolibrary of 95 in-house chalcone derivatives, identifying two hit compounds, namely, F88 and F90, as potential modulators of both transporters, whose activity on *Staphylococcus aureus* strains overexpressing NorA and resistant to ciprofloxacin was subsequently confirmed. The findings of this study are expected to guide future research towards developing novel potent chalconic inhibitors of P-gp and/or NorA.

## 1. Introduction

Multidrug resistance (MDR) is one of the main obstacles that challenge the clinical treatment of tumors and infections in humans during the last decade. Tumors and bacteria protect themselves from chemotherapeutic agents and antibiotics through different mechanisms, one of which is drug extrusion induced by membrane transport proteins known as efflux pumps [1]. In particular, the human P-glycoprotein (P-gp) and the NorA protein of *Staphylococcus aureus* (SA) are among the most researched targets, due to their vital role in transporting drugs out of cells, leading to resistance to anticancer medications and antibiotics [1–3].

In 1976, the human P-glycoprotein (P-gp) was first described as an ATP-dependent membrane transporter responsible for active drug efflux [4]. The protein, also known as ABCB1 (ATP-binding cassette subfamily B member 1) or MDR1 (multidrug resistance protein 1), is presented throughout the body, in different normal tissues such as the brain, liver, kidney, and intestines [5]. P-gp plays a crucial role in protection against xenobiotics. Nevertheless, it also negatively influences the ADMET (absorption, distribution, metabolism, excretion, and toxicity) properties of many medications as well as drug-like molecules [6]. On the other hand, the microbial efflux pump NorA is one of the major facilitator superfamily (MFS) transporters which utilize the proton gradient as an energy source to drive the extrusion of their substrates and confer MDR upon Gram-positive bacteria [7]. The activity of this protein is thought to be the cause of resistance to fluoroquinolone and ciprofloxacin reported in different strains of *S. aureus* [8–10].

The *in vitro* antimicrobial activities of natural compounds extracted from medical plants have long been confirmed [11, 12]. Tackling the multidrug resistance problem, phytochemicals would be great resistance-modifying agents, which could directly inhibit bacteria or interact with crucial factors in the pathogenesis pathway, thereby decreasing the bacteria's resistance ability. Phytochemicals might have a direct antimicrobial effect or might improve the effectiveness of antibiotics in combination treatment [13, 14]. The inhibitory activity towards four strains of *S. aureus* of caffeic acid and gallic acid was assessed in the literature [15]; these strains included the wild type (1199), the Nor-A harboring fluoroquinolone-resistant phenotype (1199B), the TetK pump possessing strain (IS-58), and the MRSA pump possessing variant (RN4220). As a result, both gallic acid and caffeic acid could lead to a reversal of antimicrobial resistance, with the latter inhibiting both MRSA and Nor-A of RN4220 and 1199B. Besides that, many previous studies have demonstrated that antibacterial resistance is significantly mediated by various natural products and semisynthetic derivatives, suggesting potential break resistance by adjuvants [16–21]. These substances showed a solid ability to increase the activity of inhibitors towards MDR bacteria, with a lower MIC of antibiotics and/or Nor-A pump substrates (such as ethidium bromide or benzalkonium chloride) upon coadministration. Thus, finding novel substances, especially natural chemicals and their derivatives that could act as Nor-A pump substrates and inhibit this reverse pump, would be a pivotal point in the fight against resistance mechanisms of bacteria.

Over many decades of research, no drugs have been approved as clinical P-gp blockers, although the use of small molecular inhibitors (SMIs) of P-gp to render tumor cells sensitive to chemotherapy has been widely acknowledged [22–25], with three generations of P-gp SMIs having been developed and tested in preclinical and clinical settings [26]. The failure of such candidates may be due to their unfavorable properties (e.g., poor solubility, poor specificity, and toxicity) and pharmacokinetic interactions [26–30]. Besides, no NorA inhibitors have been tested in humans. In subsequent research efforts, including *in silico* studies, flavonoid frameworks were also considered for the design of potential regulators of these two inverted pumps. Chemical modifications on flavonoids of herbal origins have been implemented for structure-activity relationship (SAR) studies [31]. In our 2016 publication, 87 in-house chalcones were subjected to virtual screening, during which they were docked into the ligand-binding pocket of a P-gp homology model [32]. The docking results showed good binding affinities of these molecules into the internal cavity of the protein, denoting the potential of this scaffold as a promising structure for further design of novel compounds capable of modulating this ABC efflux pump. As known regulators of P-gp and NorA have been reported to share chemical features and some of them are able to block the action of both transporters [33], it is of great interest to test the predicted P-gp-inhibiting molecules derived from the chalcone skeleton on the NorA protein, to examine whether they may exert dual activity or not.

In this study, a new chemolibrary comprising 95 in-house chalconic compounds with diverse substituents was first subject to virtual screening, using the 2-dimensional quantitative structure-activity relationship (2D QSAR) models developed and validated in our previous study [32], as well as a docking protocol employing our recently reported P-gp homology structure [32]. The predicted P-gp-regulating chalcones were then *in silico* studied, with the use of our in-house developed machine learning model and a docking procedure already published [32, 34], to predict their activity on NorA, before having their effectiveness confirmed by *in vitro* testing on different strains of *S. aureus*. The hit compounds found in this study and the remarks provided herein are expected to guide future research on novel modulators of P-gp and NorA, notably those derived from the chalcone scaffold.

## 2. Materials and Methods

### 2.1. QSAR Study

In the preparation step, the ligands' 2D structures were created in the ChemBioDrawUltra 12.0 software and then had their intramolecular energy minimized using MOE 2015.10. A total of 184 MOE 2D descriptors and 1444 PaDEL 1D and 2D descriptors representing 63 different types of molecular properties, along with 166 MACCS (Molecular ACCess System) fingerprints, 881 PubChem fingerprints, and 307 substructure fingerprints, were used to feature the molecules for subsequent construction of classification models, regression models, and cognitive map models.

First, substances with nonnull descriptive parameters were filtered out with the “Filter Examples” operated by RapidMiner 5.3.008. After that, unuseful and/or strongly correlated parameters (>0.95) were removed and the selected descriptors were optimized using RapidMiner operators. Finally, descriptive parameters were selected and cross-evaluated 10 times using the BestFirst method in Weka 3.7.9. All parameters during the variable selection process were set as default. Two tools in MOE 2015.10, “Rand” and “Diverse Subset”, were used to ensure that the data were randomly divided and substances in each data file were ranked based on the distance between each pair of them. The domain of applicability was estimated based on the theory of normalization by the “Applicability domain using standardization approach” option. From that, substances outside the domain of applicability were determined by comparing calculated descriptors with a threshold of 3 sigma. Machine learning methods by Clementine 12.0 were implemented, including the ganglia binary classifier and ensemble for classification based on P-gp-inhibiting and non-P-gp-inhibiting ligands to predict the activity on this reverse pump. The selected default conditions of each operator in the ganglia were estimated through single machine models: neural network; C5.0; classification and regression tree (C&R Tree); quick, unbiased, efficient, and statistical tree (QUEST); chi-square automatic interaction detector (CHAID); logistic regression; decisive tilt; Bayesian network; linear discriminant analysis; and support vector machine (SVM).

Classification models were evaluated by four metrics including true positives (TP), false positives (FP), true negatives (TN), and false negatives (FN). Regression models were evaluated through statistical metrics to measure the predictability of QSAR models. The quality of models was evaluated by the correlation coefficient *R*^2^ and the cross-validation correlation coefficient *Q*^2^. Parameters and the concordance correlation coefficient (CCC) were applied for the external evaluation of models when substances not related to model development were predicted.

### 2.2. Docking

A subsequent molecular docking study on the aforementioned chalcone derivatives was carried out, using a homology model of human P-gp developed from a crystallographic structure of the same transporter found in *Mus musculus*/house mouse (PDB ID 3g61) and the same docking protocol that was reported in our previous study [32]. The predicted ligand-binding site located at the interface of the transmembrane domains TM3 and TM11, as indicated in our 2016 paper and in agreement with results from other researchers (published in 2006 and 2009), was used as the docking site [32, 35, 36].

A combination of ligand-based and structure-based methods was used to build different *in silico* models. In the data preparation step, all ligands' 2D structures were created by ChemBioDrawUltra 12.0 and then had their energy minimized by MOE 2015.10, while the selected homology protein was prepared by the LigX tool in MOE. The protein's binding site was predicted by the I-TASSER server and was then the area into which all compounds were docked to identify potential candidates through ligand-protein interactions. The triangle matching theory algorithm was used with a maximal number of 1000 solutions per iteration and 200 solutions per fragmentation [34, 37–39].

### 2.3. Biotesting

In this study, potential substances were tested in terms of ability to reduce the resistance of bacterial strains toward antibiotics. Specifically, the selected in-house chalcones were combined with ciprofloxacin, an antibiotic which *S. aureus* has been reported to resist due to the reverse pump NorA. A total of seven *S. aureus* strains were involved in this part of the study, including SA-1199 (wild-type strain without NorA overexpression isolated from sepsis patients), SA-1199B (mutant strain with NorA overexpression isolated from rabbit endocarditis models and provided by Pr. Rybak M. J., Wayne State University, USA) [40, 41], and five clinically observed ciprofloxacin-resistant strains isolated from medical waste. The presence of NorA in the collected test samples and its implication in ciprofloxacin resistance were confirmed by the suppliers.

The bioactivity of the tested molecules was evaluated using the dilution test as described in the literature [42, 43] and based on the minimal inhibitory concentration (MIC, in *μ*g/ml) of ciprofloxacin when used alone and in combination with each of the chalcones on all aforementioned strains of *S. aureus*. More specifically, the substances were diluted in a defined volume of a suitable solvent, 10 *μ*l of this original solution was then combined with 5 ml of the Mueller-Hinton broth (MHB) environment. This MHB tube then received 128 *μ*l of a solution of ciprofloxacin (2 mg/ml), giving a solution of ciprofloxacin at the exact concentration of 256 *μ*g/ml (Ci). The tested bacteria were cultured on tryptone soya agar (TSA) and incubated at 37°C for 24 hours. Subsequently, 3-5 colonies were taken and transferred to a tryptone soya broth (TSB) medium. The incubation process took place during 2-6 hours at 37°C. The microbial optical density (OD) was adjusted with physiological saline to obtain an equivalent standard McFarland turbidity level of 0.5 (approximately 1.5 × 10^8^ CFU/ml). The bacterial suspension continued to be diluted 100 times and would be ready for use in 15 minutes. All media were sterilized at 121°C and 1 atm for 20 minutes before use and conserved at 2-8°C.

During the evaluation, the results were only valid if the bacteria in the 12th well could grow normally. The MIC values of ciprofloxacin on SA-mutant strains with the presence of the tested chalcones were compared to that of ciprofloxacin when used alone.

### 2.4. Chemical Synthesis

**Scheme 1:**
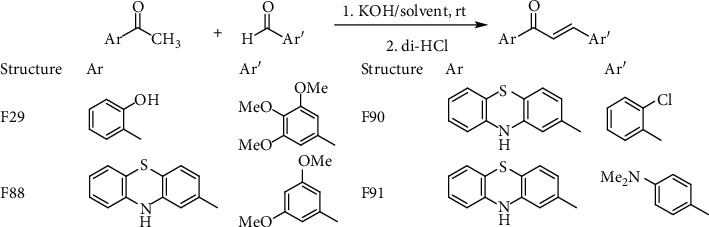
General key step for the synthesis of the four selected chalcones.

The synthesis of four new chalcones, namely, F29, F88, F90, and F91, which were reported in previous studies [44–46], was based on the classical Claisen-Schmidt condensation. This reaction is an aldol condensation process between an acetophenone derivative (forming the A ring of the product) and an aryl aldehyde (serving as the B ring) in methanol/KOH at room temperature (Scheme 1). Specifically, the chalcones F88, F90, and F91 were synthesized by replacing the phenyl A ring with a phenothiazine ring, while the compound F29 was synthesized by adding substituents on both rings.

The physicochemical properties and spectral characteristics of these hits are shown as follows.

- F29: *IUPAC Name*: (E)-1-(2-hydroxyphenyl)-3-(3,4,5-trimethoxyphenyl)prop-2-en-1-one. *Yield*: 55%. *Melting point*: 156-158°C. *UV (λ*_max_*nm, MeOH)*: 363; 251. *IR (KBr) cm^−1^*: 1635.5; 1568.0; 1155.4. *HR-MS (ESI)*: [M+H]^+^*m*/*z* = 315.1235. *^1^H-NMR (DMSO-d6)δppm*: 8.25–8.24 (*d*, 1H, *J* = 8 Hz, H6′); 7.90 (*s*, 2H, H*α* và H*β*); 7.57–7.54 (*t*, 1H, *J* = 7.5 Hz, H5′); 7.16 (*s*, 2H, H2 và H6); 7.04–7.00 (*t*, 1H, *J* = 7.5 Hz, H4′); 7.02–7.00 (*d*, 1H, *J* = 9.5 Hz, H3′); 3.95 (*s*, 6H, Ar-OCH_3_); 3.85 (*s*, 3H, Ar-OCH_3_). *^13^C-NMR (DMSO-d6)δppm*: 193.7, 162.1, 153.1 (2 × *C*), 145.5, 140.2, 136.2, 130.8, 129.9, 120.6, 120.5, 118.9, 117.7, 106.9 (2 × *C*), 60.1, 56.1 (2 × *C*)
- F88: *IUPAC Name*: (E)-3-(2-chlorophenyl)-1-(10H-phenothiazin-2-yl)prop-2-en-1-one. *Yield*: 57%. *Melting point*: 202-203°C. *EIMSm*/*z*: 386.0331 [M+Na]^+^. *UV (λ*_max_*nm, MeOH)*: 204, 248, 309, 449. *IR (KBr) cm^−1^*: 3354, 1654, 1590, 754. *^1^H-NMR (DMSO)δppm*: 8.79 (*s*, 1H, NH); 8.16 (*d*, *J*_3″−4″_ = 7.5 Hz, 1H, H3^″^); 8.00 (*d*, *J*_3−2_ = 15.5 Hz, 1H, H3); 7.84 (*d*, *J*_2−3_ = 15.5 Hz, 1H, H2); 7.64 (*d*, *J*_3′−4′_ = 8 Hz, 1H, H3′); 7.57 (*d*, *J*_6″−5″_ = 7.5 Hz, 1H, H6^″^); 7.46 (*m*, 2H, H4^″^, H5^″^); 7.30 (*s*, 1H, H1′); 7.09 (*d*, *J*_4′−3′_ = 8 Hz, 1H, H4′); 7.01 (*m*, 1H, H8′); 6.92 (*d*, *J*_6′−7′_ = 7.5 Hz, 1H, H6′); 6.66 (*m*, 1H, H7′); 6.66 (*d*, *J*_9′−8′_ = 8 Hz, 1H, H9′). *^13^C-NMR (DMSO)δppm*: 187.7 (C1 = O); 142.1 (C3); 141.0 (C13′); 138.2 (C11′); 136.5 (C2′); 134.2 (C2^″^); 132.2 (C1^″^); 131.9 (C3^″^); 130 (C4^″^); 128.4 (C8′); 127.9 (C6^″^); 127.6 (C5′); 126.2 (C5^″^); 126.1 (C4′); 124.5 (C7′); 124.4 (C2); 122.7 (C3′); 115.1 (C12′); 114.5 (C14′); 112.8 (C9′)
- F90: *IUPAC Name*: (E)-3-(2,4-dimethoxyphenyl)-1-(10H-phenothiazin-2-yl)prop-2-en-1-one. *Yield*: 49%. *Melting point*: 178-179°C. *EIMSm*/*z*: 388.1154 [M–H]^−^. *UV (λ*_max_*nm, MeOH)*: 204, 248, 304, 365*. IR (KBr) cm^−1^*: 3310, 1643, 1570, 1272, 1150. *^1^H-NMR (DMSO)δppm*: 8.76 (*s*, 1H, NH); 7.95 (*d*, *J*_3−2_ = 15.5 Hz, 1H, H3); 7.86 (*d*, *J*_6″−5″_ = 8.5 Hz, 1H, H6^″^); 7.61 (*d*, *J*_2−3_ = 15.5 Hz, 1H, H2); 7.52 (*dd*, *J*_3′−4′_ = 8 Hz, *J*_3′−1′_ = 1.5 Hz, 1H, H3′); 7.28 (*d*, *J*_1′−3′_ = 1.5 Hz, 1H, H1′); 7.06 (*d*, *J*_4′−3′_ = 8 Hz, 1H, H4′); 7.01 (*m*, 1H, H8′); 6.92 (*d*, *J*_6′−7′_ = 7 Hz, 1H, H6′); 6.80 (*m*, 1H, H7′); 6.64 (*m*, 3H, H9′, H3^″^, H5^″^); 3.90 (*s*, 3H, 2^″^-OMe); 3.84 (*s*, 3H, 4′-OMe). *^13^C-NMR (DMSO)δppm*: 187.9 (C1 = O); 163.1 (C4^″^); 160 (C2^″^); 142.1 (C13′); 141.2 (C11′); 138.6 (C3); 137.3 (C2′); 130.1 (C6^″^); 127.9 (C8′); 126.2 (C6′); 126.1 (C4′); 123.0 (C7′); 122.1 (C2); 122.0 (C3′); 118.9 (C1^″^); 115.9 (C1′); 115.2 (C12′); 114.5 (C14′); 112.9 (C9′); 106.3 (C5^″^); 98.3 (C3^″^); 55.8 (2′-MeO); 55.5 (4′-CH_3_O)
- F91: *IUPAC Name*: (E)-3-(4-(N,N-dimethylamino)phenyl)-1-(10H-phenothiazin-2-yl)prop-2-en-1-one. *Yield*: 45%. *Melting point*: 232-233°C. *EIMSm*/*z*: 371.1286 [M–H]^–^. *UV (λ*_max_*nm, MeOH)*: 204; 248; 292; 425. *IR (KBr) cm^−1^*: 3314; 1650; 1600. *^1^H-NMR (DMSO)δppm*: 8.75 (*s*, 1H, NH), 7.67–7.64 (*d*, 1H, *J*_3–2_ = 15.5 Hz, H3); 7.65–7.63 (*d*, 2H, *J*_2″–3″_ = *J*_6″–5″_ = 9 Hz, H2^″^, H6^″^); 7.55 (*d*, *J*_3′–4′_ = 7.5 Hz, 1H, H3′); 7.47 (*d*, *J*_2–3_ = 15.5 Hz, 1H, H2); 7.30 (*s*, 1H, H1′); 7.05 (*d*, *J*_4′–3′_ = 7.5 Hz, 1H, H4′); 7.00 (*t*, *J*_8′–7′_ = *J*_8′–9′_ = 7.5 Hz); 6.92 (*d*, *J*_6′–7′_ = 7.5 Hz, 1H, H6′); 6.76 (*t*, *J*_7′–6′_ = 7.5 Hz, 1H, H7′); 6.74 (*d*, *J*_3″–2″_ = 9 Hz, *J*_5″–6″_ = 9 Hz, 2H, H3^″^, H5^″^); 6.67 (*d*, *J*_8.1−9.1_ = 7.5 Hz, 1H, H9′); 3.00 (*s*, 6H, NMe2). ^13^C-*NMR (DMSO)δppm*: 187.4 (C1 = O); 151.9 (C4^″^); 144.9 (C3); 142.0 (C13′); 141.2 (C11^″^); 137.7 (C2′); 130.6 (C2^″^, C6^″^); 127.9 (C8′); 126.2 (C6′); 126.0 (C4′); 122.5 (C1^″^); 122.0 (C7′); 122.0 (C2); 121.9 (C3′); 115.8 (C1′); 115.3 (C12′); 114.5 (C14′); 112.9 (C9′); 111.7 (C3^″^, C5^″^); 39.63 (NMe2)

## 3. Results and Discussion

### 3.1. Results

#### 3.1.1. In Silico Screening for Chalconic Inhibitors of P-gp Using 2D QSAR and Molecular Docking

On the basis of six machine learning 2D QSAR models for predicting P-gp inhibitory activity of chalcone derivatives, an ensemble model was designed by combining the aforementioned individual models. The ensemble model achieved good performances on both the training and validation datasets, and a higher overall accuracy level was reported in our seminal paper in 2016 [32]. In this study, this model was applied to a novel dataset composed of 95 in-house designed chalcone derivatives, among which 27 molecules were predicted as potential regulators of P-gp, as their calculated IC50 values were below 15 *μ*M, the threshold to distinguish P-gp inhibitors from noninhibitors [47]. Interestingly, these chalcones were also deemed P-gp modulators by most of the machine learning classification models used for predicting regulators of this efflux pump that were reported in our previous study on this topic [48], denoting a high agreement level of these virtual screening tools on selecting potential inhibitors of P-gp from a pool of different chalconic compounds. The 2D structures of these 27 derivatives are provided in Figure S1.

**Figure 1:**
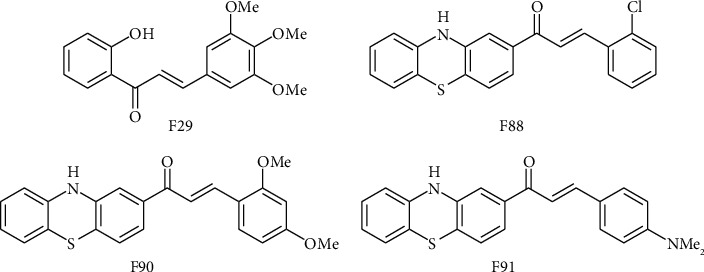
Two-dimensional structures of F29, F88, F90, and F91, which are the in-house chalcones predicted as P-gp inhibitors by our 2D QSAR model and docking protocol.

Docking results, in combination with 2D QSAR screening outcomes indicated above, led to the selection of four chalcone derivatives, namely, F29, F88, F90, and F91 (Figure 1) for the next step of this study. These molecules gave good predicted IC50 values (≤15 *μ*M), were deemed potential P-gp modulators by almost all classification models, and had relatively strong binding affinities with the binding pocket, with docking scores ranging from -21.52 kJ mol^−1^ to -15.38 kJ mol^−1^, comparable to or even better than those obtained from known P-gp inhibitors such as reserpine (-3.47 kJ mol^−1^), elacridar (-22.86 kJ mol^−1^), tariquidar (-13.26 kJ mol^−1^), and saquinavir (-19.31 kJ mol^−1^) [49, 50]. An analysis of these molecules' docking poses inside the active site points out the amino acids His61, Leu65, Phe194, Gln195, Gln946, Met949, and Tyr950 as key residues responsible for forming intermolecular interactions, including hydrogen bonds, between the small ligands and the macromolecule, once again confirming earlier results regarding the composition of this transporter's ligand-binding site [36].

#### 3.1.2. In Silico Study on the Four Selected Chalcone Derivatives and In Vitro Testing of Their Activity as Potential NorA Inhibitors

A linear regression model constructed by the partial least square method, as reported in our previous paper, was employed for this step of the study [24, 32]. The model uses three different descriptors that take into account both physicochemical and topological characteristics of small molecules to predict their potency on the NorA protein, as portrayed in Equation (1). The four chalcone derivatives (F29, F88, F90, and F91), which were previously deemed the most potential P-gp modulators out of the 95 in-house designed chalconic compounds, had their pIC50 values (-logIC50) against NorA calculated by Equation (1) to examine whether they are also capable of inhibiting this transporter, due to the fact that the known ligands of these two efflux pumps are reported to have many chemical features in common and that their structures have a considerable degree of overlap [33]. Besides, a homology model of NorA developed from a published structure of the multidrug transporter EmrD found in *E. coli* (PDB ID 2gfp), which belongs to the same major facilitator superfamily as NorA, was employed for molecular docking in this study [32, 34]. The two ligand-binding pockets located at the central channel and the Walker B motif of the transporter were used as docking sites. The same docking protocol described in our earlier paper was reused for this step [32, 37].

(1) $$\begin{array}{c} \text{pIC}50=5.91838–0.11195x\left(\text{rings}\right)–1.38078x\left(\text{balaban}J\right)–0.24425x\left(\log S\right). \end{array}$$

**Figure 2:**
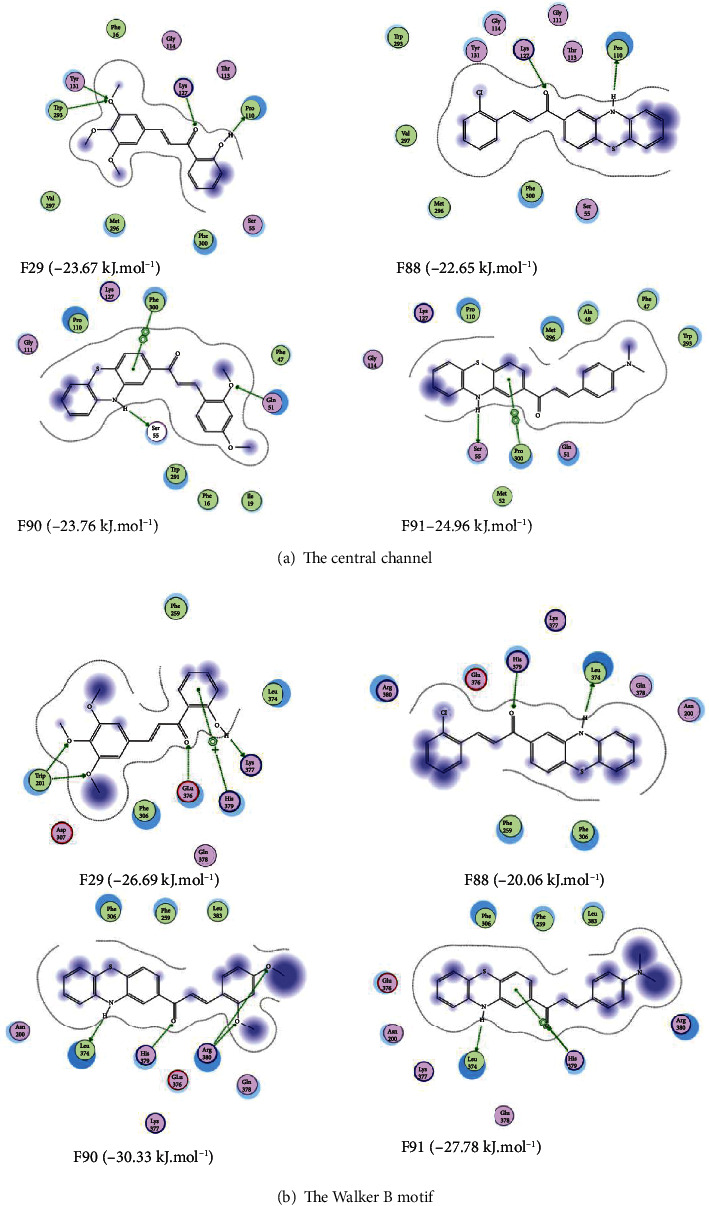
Two-dimensional interaction schemes inside the two NorA ligand-binding sites: the central channel (a) and the Walker B motif (b) of the four selected chalcones F29, F88, F90, and F91.

Results from the regression model suggested the potential of three chalcones, namely, F88, F90, and F91, as NorA inhibitors (predicted IC50 values at 3.84, 5.24, and 5.07 *μ*M, respectively). F29, on the contrary, received poor potency prediction, with theoretical IC50 calculated at 88.27 *μ*M. Docking results generally agreed with the aforementioned results, with the docking scores of F29 inside the two binding pockets denoting poorer binding affinities of this molecule with the protein in comparison to those obtained from the other three compounds in most cases (Table 1). The key amino acid residues responsible for intermolecular interactions between the small ligands and the binding sites are Gln51, Ser55, Pro110, Lys127, Tyr131, Trp293, Met296, Val297, and Phe300 (at the central channel) and Asn200, Phe259, Phe306, Leu374, Glu376, Lys377, Gln378, His379, and Arg380 (at the Walker B motif). All details in this regard can be found in Figure 2.

*In vitro* testing on the ability of these four chalcones to hamper the action of NorA was carried out. Results are portrayed in Table 2.

### 3.2. Discussions

#### 3.2.1. In Silico Study

The results of *in silico* screening showed a similar prediction in all models, in which F89, F90, and F91 were the three most suitable P-gp inhibitors, and these chalcones may also inhibit the Nor-A protein as well. In addition, F88 could be more potential in NorA inhibition, as predicted by IC50 regression models and previously described pharmacophore models. *In silico* screening models were also applied to a Nor-A homology protein, revealing that the four chalcones, namely, F29, F88, F90, and F91, strongly contacted with NorA in the binding site. However, only F88, F90, and F91 were less soluble in water and were deemed possible for further research.

#### 3.2.2. Bioactivity Testing

As expected, ciprofloxacin itself was effective on the wild-type *S. aureus* strain SA-1199 (without NorA expression). On the NorA-overexpressed strain SA-1199B and the five resistant strains observed in clinical settings, the MICs of ciprofloxacin significantly increased, denoting a drastic drop in its effectiveness. The presence of the chalcone derivatives generally helped lower the recorded MICs of the antibiotic, with F88 improving *S. aureus* inhibition in all six strains, F90 in four strains, and F91 and F29 in two strains, suggesting that these chalcones did impede the NorA transporter, thus enhanced the bioactivity of ciprofloxacin. These findings generally agreed with the aforementioned *in silico* results, confirming the inferiority of F29 as a NorA regulator and highlighting the potential of two particular compounds, namely, F88 and F90 as candidates for novel molecules capable of inhibiting NorA. These two hits can be easily synthesized with a high purity level, via the Claisen-Schmidt condensation reaction at room temperature during several hours, using 2-acetylphenothiazine and appropriate aryl aldehydes, which are all commercially available, according to a protocol reported in our 2012 paper [51]. Details regarding physicochemical properties and spectral characteristics of the four selected chalcones can be found in Materials and Methods.

The above results from *in silico* and *in vitro* experiments on 95 in-house chalcone derivatives and more specifically on the four molecules, F29, F88, F90, and F91, suggest that (i) the ring A in the structure of chalcones, when substituted with a hydroxyl group at the *ortho* position, or when replaced by the phenothiazine moiety, led to better inhibitory activity against both P-gp and NorA, as more hydrogen bonds between the small molecules and the active sites would be observed, with the –OH and –NH– groups acting as hydrogen bond donors, thus contributing to the binding affinities with the binding pockets of both proteins; (ii) the methoxy and halogen substituents (in particular, chloro) on the ring B seemed preferable to more polar groups such as dimethylamino when it comes to designing chalconic NorA regulators, as the latter resulted in weaker protein-ligand interactions and poorer permeability of the molecules across the lipid membrane; and (iii) the presence of substituents at the *para* position on the ring B seemed more necessary for improving the bioactivity of P-gp modulators rather than for that of NorA inhibitors. More *in silico* and *in vitro* research in this regard is encouraged to be carried out, taking into account the results portrayed in this study, to further validate the points raised herein and to design new regulators of P-gp and/or NorA, in hopes of alleviating antibiotic resistance of bacterial strains overexpressing these efflux pumps. Such compounds may mimic the structures of our chalcone derivatives or those of classic efflux pump inhibitors and/or substrates such as carbonyl cyanide m-chlorophenyl hydrazone (CCCP), ethidium bromide, or benzalkonium chloride.

## 4. Conclusions

In brief, finding novel molecules capable of inhibiting the action of efflux pumps implicated in antibacterial resistance is a task that has long been tackled by medicinal chemists. In this study, an application of our earlier published work, including machine learning models for predicting P-gp modulators and NorA regulators, as well as two homology 3D structures of these two proteins, is described. The outcome of this present study provides two chalcone derivatives, F88 and F90, as potential inhibitors of both transporters, with effectiveness on different *Staphylococcus aureus* strains overexpressing NorA and resisting ciprofloxacin confirmed by *in vitro* experiments. More biological testing on these two “hits” is expected to be carried out, more specifically, with bacterial strains and/or tumor cells where an overexpression of P-gp and/or NorA is observed, to fully validate their bioactivity. Further optimization of these “hits” is also encouraged, taking inspiration from the remarks indicated above and employing our computational models, with a view to confirming the potential of the chalcone skeleton as a privileged structure for designing novel efflux pump modulators, and increasing the sensitivity of bacterial strains to antibiotics, as well as of tumor cells to chemotherapy.

## Figures and Tables

**Table 1:** Predicted IC_50_ values (*μ*M) on NorA and docking scores (kJ mol^−1^) of the four selected in-house chalcones inside the binding sites.

Chalcone	Predicted IC_50_	Docking score (kJ mol^−1^)	
		Central channel	Walker B motif
F29	88.27	-23.67	-26.69
F88	3.84	-22.65	-29.06
F90	5.24	-23.76	-30.33
F91	5.07	-24.96	-27.78

**Table 2:** MIC values (*μ*g/ml) of ciprofloxacin (Ci) and synthesized chalcones when used alone and in combination with each of the chalcones on different strains of *S. aureus*.

*S. aureus* strain	MIC (*μ*g/ml)								
	Ci	F29	F88	F90	F91	Ci+F29	Ci+F88	Ci+F90	Ci+F91
SA-1199^†^	<0.125	512	512	512	512	<0.125	<0.125	<0.125	<0.125
SA-1199B^†^	4	>512	>512	>512	>512	4	2	2	4
I16.1421^‡^	0.5	512	256	512	512	0.125	0.125	0.125	0.5
I16.1505^‡^	32	512	512	512	512	32	16	32	32
I16.1562^‡^	32	>512	256	>512	>512	32	16	32	32
I16.1635^‡^	32	256	>512	256	>512	32	16	16	16
I16.1672^‡^	32	>512	512	512	>512	16	16	16	16

Concentrations of the chalcones: ^†^50 *μ*g/ml and ^‡^20 *μ*g/ml.

## Data Availability

Supplementary material is provided as a PDF file containing the following information: Figure S1: 2D structures of 27 chalcone derivatives predicted as potential P-gp inhibitors by our 2D QSAR model. Figure S2: IR spectra of the four selected chalcones F29, F88, F90, and F91. Supporting information is available free of charge.

## Supplementary Materials

Supplementary Materials Supplementary material is provided as a PDF file containing the following information: Figure S1: 2D structures of 27 chalcone derivatives predicted as potential P-gp inhibitors by our 2D QSAR model. Figure S2: IR spectra of the four selected chalcones F29, F88, F90, and F91. Supporting information is available free of charge.
