# Supplementary material for: Chalcone Derivatives as Potential Inhibitors of P-Glycoprotein and NorA: An In Silico and In Vitro Study
Source: Biomed Res Int. 2022 Mar 26;2022:9982453. doi: 10.1155/2022/9982453 (PMC8976639; doi:10.1155/2022/9982453)
Supplement: Supplementary Materials — Supplementary material is provided as a PDF file containing the following information: Figure S1: 2D structures of 27 chalcone derivatives predicted as potential P-gp inhibitors by our 2D QSAR model. Figure S2: IR spectra of the four selected chalcones F29, F88, F90, and F91. Supporting information is available free of charge. [file 9982453.f1.docx]

**Supplementary Material**

**Chalcone derivatives as potential inhibitors of P-glycoprotein and NorA: an *in silico* and *in vitro* study**

Minh-Tri Le,^1,2,#^ Dieu-Thuong Thi Trinh^3,4,#^, Trieu-Du Ngo,^1^ Viet-Khoa Tran-Nguyen,^1^ Dac-Nhan Nguyen,^1^ Tung Hoang,^1^ Hoang-Minh Nguyen,^1^ Tran-Giang-Son Do,^1^ Thanh-Tan Mai,^1^ Thanh-Dao Tran,^1,*^ and Khac-Minh Thai^1,*^

^1^Department of Medicinal Chemistry, Faculty of Pharmacy, University of Medicine and Pharmacy at Ho Chi Minh City, 41 Dinh Tien Hoang, Dist 1, Ho Chi Minh City 700000, Vietnam

^2^School of Medicine, Vietnam National University Ho Chi Minh City, Linh Trung Ward, Thu Duc District, Ho Chi Minh City, 700000 Vietnam

^3^Faculty of Traditional Medicine, University of Medicine and Pharmacy at Ho Chi Minh City, 700000, Vietnam

^4^University Medical Center Ho Chi Minh City, University of Medicine and Pharmacy at Ho Chi Minh City, 700000, Vietnam.

# These authors contributed equally to this work and are co-first authors.

Correspondence should be addressed to Thanh-Dao Tran (daott@ump.edu.vn) and Khac-Minh Thai (thaikhacminh@ump.edu.vn; thaikhacminh@gmail.com)

|  |  |  |
| --- | --- | --- |
| F4 | F5 | F6 |
|  |  |  |
| F7 | F11 | F14 |
|  |  |  |
| F16 | F17 | F18 |
|  |  |  |
| F29 | F30 | F32 |
|  |  |  |
| F33 | F34 | F35 |
|  |  |  |
| F36 | F37 | F44 |
|  |  |  |
| F45 | F61 | F63 |
|  |  |  |
| F65 | F88 | F89 |
|  |  |  |
| F90 | F91 | F95 |

**Figure S1.** 2D structures of 27 chalcone derivatives predicted as potential P-gp inhibitors by our 2D QSAR model.

**F29**


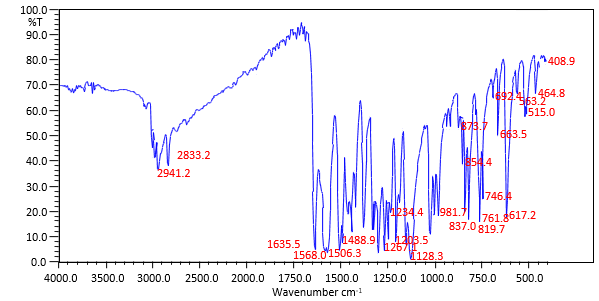


**F88**

**
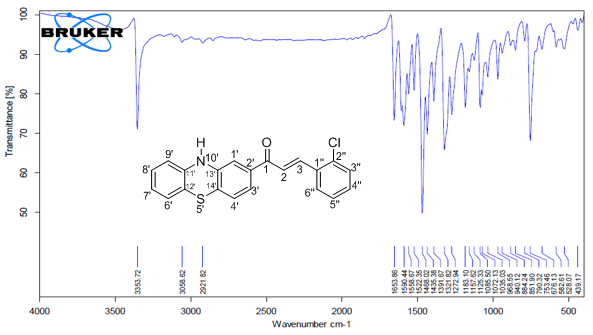
**

**F90**

**
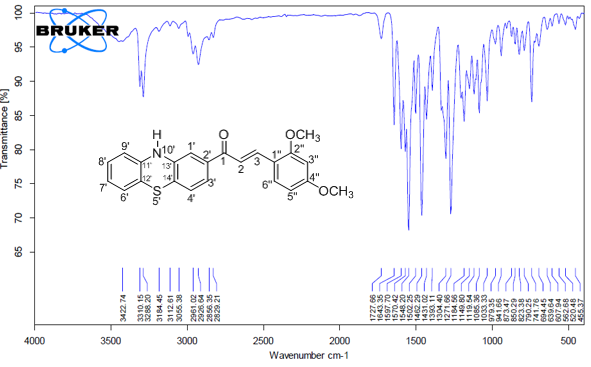
**

**F91**

**
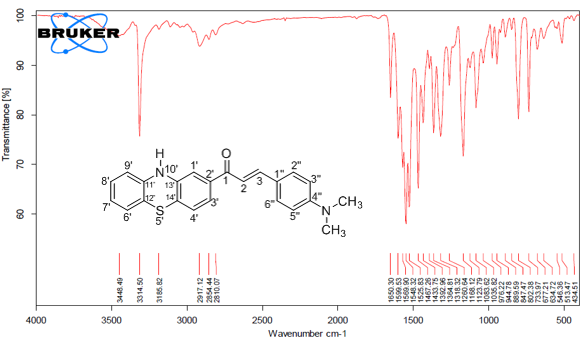
**

**Figure S2.** IR-spectra of the four selected chalcones F29, F88, F90 and F91.
